# Toward a Unifying Account of Dopamine’s Role in Cost-Benefit Decision Making

**DOI:** 10.1016/j.bpsgos.2022.02.010

**Published:** 2022-03-08

**Authors:** Alexander Soutschek, Alexander Jetter, Philippe N. Tobler

**Affiliations:** a Department of Psychology, Ludwig Maximilian University Munich, Munich, Germany; b Department of Clinical Pharmacology and Toxicology, University Hospital Zürich, University of Zürich, Zürich, Switzerland; c Zürich Center for Neuroeconomics, Department of Economics, University of Zürich, Zürich, Switzerland; d Neuroscience Center Zürich, Swiss Federal Institute of Technology Zürich, University of Zürich, Zürich, Switzerland

**Keywords:** Delay discounting, Dopamine, Effort discounting, Psychopharmacology, Reward, Risky decision making

## Abstract

Dopamine is thought to play a crucial role in cost-benefit decision making, but so far there is no consensus on the precise role of dopamine in decision making. Here, we review the literature on dopaminergic manipulations of cost-benefit decision making in humans and evaluate how well different theoretical accounts explain the existing body of evidence. Reduced D_2_ stimulation tends to increase the willingness to bear delay and risk costs (i.e., wait for later rewards, take riskier options), while increased D_1_ and D_2_ receptor stimulation increases willingness to bear effort costs. We argue that the empirical findings can best be explained by combining the strengths of two theoretical accounts: in cost-benefit decision making, dopamine may play a dual role both in promoting the pursuit of psychologically close options (e.g., sooner and safer rewards) and in computing which costs are acceptable for a reward at stake. Moreover, we identify several limiting factors in the study designs of previous investigations that prevented a fuller understanding of dopamine’s role in value-based choice. Together, the proposed theoretical framework and the methodological suggestions for future studies may bring us closer to a unifying account of dopamine in healthy and impaired cost-benefit decision making.

In everyday life, we often face tradeoffs between desired goods (e.g., eating a chocolate cake, consuming drugs) and the nondesired costs one has to bear to obtain those goods (gaining weight, long-term consequences of drug abuse). These costs come in different forms such as delay of reward delivery, risk or ambiguity, mental or physical effort, or social costs such as inequity (1). Deficits in cost-benefit decision making belong to the core symptoms of several psychiatric disorders, including depression, schizophrenia, addiction, eating disorders, gambling disorders, or Parkinson’s disease (2, 3, 4, 5, 6). Substance addiction, for example, can be understood as maladaptive preference for immediate reinforcement (acute drug effects) under ignorance of negative long-term consequences (2). Insights into the neurochemical basis of cost-benefit decisions may therefore improve our understanding of the neural origins of decision-making deficits in psychiatric disorders as well as the effectiveness of pharmacological treatments.

A large body of evidence assigns a crucial role to the neurotransmitter dopamine in weighing costs against benefits [for a recent review, see (7)]. However, there is no agreement on the theoretical interpretation of these empirical findings, because several accounts with different hypothesized roles for dopamine in cost-benefit decision making have been postulated. Often, the predictions of different accounts are even in apparent conflict with each other: for example, while some accounts claim that dopamine increases the tolerance for delay costs (8,9), others posit that dopamine enhances the preference for immediate rewards (10,11). These conflicting assumptions hamper both the development of pharmacological treatments of decision-making deficits and the prediction of the impact of dopaminergic treatment on decision making. Thus, it is of major importance to develop a coherent account of dopamine’s function in cost-benefit decision making.

This review has three main goals. First, we assess whether prominent accounts of dopaminergic contributions to decision making can explain existing empirical findings. Each of the existing accounts can explain only a subset of the data, and we argue that none of them provides a coherent picture of the role of dopamine in cost-benefit weighing. Second, based on this, we aim to outline a model for dopamine in value-based choice, which combines the strengths of existing accounts. Third, we argue that the development of a coherent theoretical account is hampered by limitations in the design of decision neuroscience studies and make suggestions about which kind of future research can facilitate arbitration between different theoretical models.

## Empirical Findings for Dopaminergic Manipulations in Decision Making

We first summarize the empirical literature on the influences of dopaminergic pharmacological manipulations on cost-benefit decision making in healthy volunteers. We separately discuss four different cost types: delay, risk, effort, and social costs. We also consider which dopamine receptor subtypes are targeted by a given pharmacological manipulation because some of the accounts described below (see Relating Empirical Findings to Existing Accounts of Dopamine Functioning in Value-Based Choice) suggest that different receptor subtypes play dissociable roles in cost-benefit decision making. In particular, dopamine receptors can be subdivided into D_1_-type receptor (D1R) and D_2_-type receptor (D2R) families. D1Rs are prevalent in the striatal direct “go” path, which is thought to energize behavior toward goals (12). D2Rs, in contrast, are expressed predominantly in the indirect “no go” path, which is involved in action inhibition. Because D2R activation suppresses activation in the inhibitory indirect path, stronger D2R signaling also energizes behavior by suppressing the inhibitory influence of the indirect path on cortical activity (13,14).

We first consider tradeoffs between reward magnitudes and temporal delays to reward delivery, as studied in intertemporal choices where agents typically have to choose between a smaller-sooner (e.g., 30 Swiss francs today) and a larger-later reward (e.g., 100 Swiss francs in 100 days) (Table S1). The majority of pharmacological studies used D_2_ antagonists or agents increasing both D1R and D2R activity (such as levodopa or d-amphetamine). Of the five studies employing selective D_2_ antagonists, four observed a stronger preference for delayed rewards after D2R blockade (15, 16, 17, 18), while one study observed no significant effects (10). Similarly, an unspecific decrease in dopamine transmission through acute phenylalanine and tyrosine depletion had no effect on intertemporal choice (19). Overall (and under the assumption that the used antagonists act post- rather than presynaptically), the data suggest that reduction of D2R activity reduces delay costs.

Compounds unspecifically increasing D1R and D2R activity showed mixed effects, i.e., either no impact on intertemporal decision making (20,21); a stronger preference for delayed rewards, at least at higher doses (8,9); or a reduced preference for delayed rewards (10). In addition, selective D_1_ or D_2_ agonists revealed no significant effects (22,23). The lack of consistent significant effects of dopamine agonists might be explained by inverted u–shaped response curves where dopamine agonists, particularly at higher doses, increase dopaminergic activity beyond the optimal performance level for some but not other individuals. Thus, the only relatively robust finding in the domain of waiting costs is that selective reduction of D2R neurotransmission decreases the preference for smaller-sooner rewards.

The literature on dopamine in risky decision making paints a picture that is somewhat harder to interpret (Table S2). An example for risky choices are lottery choices where agents decide between two lotteries with varying probabilities of winning or losing rewards. Dopamine agents stimulating both D1R and D2R show either no significant effects (8,20,24,25) or a higher risk tolerance, particularly in the gain domain (26, 27, 28, 29, 30). In contrast, one study with the combined (indirect) D_1_/D_2_ agonist levodopa observed a reduced preference for risky outcomes, but only in individuals with high baseline impulsivity (21). Moreover, high doses of a selective D_1_ agonist on average reduced preference for riskier but larger outcomes (22). D_2_ agonists showed no significant effects (23), increased propensity for risky choices (31), or increased sensitivity for gains combined with a lower sensitivity for losses in individuals with low reward sensitivity (32). D2R blockade showed either no significant effects (18,33) or reduced sensitivity to costs (34, 35, 36). Thus, if anything, reduced D2R neurotransmission increases the preference for high reward–high costs options, similar to the findings for delay costs.

Next, we consider tradeoffs between rewards and the costs of (mental or physical) effort (Table S3). For this cost type, pharmacological studies paint a relatively coherent picture. Dopamine agonists increase the willingness to exert effort for rewards (22,37, 38, 39, 40), with only one study showing no significant effects (41). Conversely, most dopamine antagonist studies report reduced willingness to exert effort (42, 43, 44). We note that two studies using a low dose of the D_2_ antagonists haloperidol and sulpiride (37,40) observed reduced effort discounting, consistent with the view that low doses of D_2_ antagonists increase dopaminergic activity via presynaptic effects. One study directly comparing the effects of methylphenidate and sulpiride observed that methylphenidate (stimulating both D1Rs and D2Rs) increased the sensitivity to rewards, whereas sulpiride (as selective D_2_ antagonist) attenuated the impact of costs on decision making (37). This is consistent with dissociable roles of D1Rs and D2Rs for benefit and cost processing, respectively. Taken together, pharmacological evidence on effort discounting suggests that dopamine enhances the motivation to work for rewards, with potentially dissociable roles for D1Rs and D2Rs.

Finally, we consider cost-benefit tradeoffs in social decision making (Table S4). Intuitively, one might assume that the cost of sharing goods with others opposes the goal of maximizing one’s selfish payoff, but many individuals perceive sharing as rewarding [“warm glow” effect (45,46)], and this effect seems to be stronger in women than in men (47). Correspondingly, reduced D2R neurotransmission shows gender-specific effects, with lower D2R activity reducing costly sharing in women and increasing it in men (16), particularly for close others. The same effect was observed for female participants under a D_2_ agonist (48), which may be reconciled with other findings (16) by assuming an inverted u–shaped dose-response curve [although one could alternatively assume that the D_2_ antagonist in (16) had stronger presynaptic than postsynaptic effects]. Levodopa increased selfishness in mixed samples of male and female participants (49) and in a male-only sample (50), the latter being consistent with our findings reported in (16). Methylphenidate showed no significant effect on prosocial giving in a sample of mixed female and male participants. Independently of gender, the D_1_/D_2_ agonist tolcapone increased the perceived costs of unequal outcomes (51), hinting to a role of dopamine in encoding social norms. Taken together, the effects in the social domain are difficult to interpret, particularly because it is hard to define subjective costs and benefits in social decision making (which may vary between individuals). However, under the assumption of the hypothesized gender-specific role of dopamine in social preferences, the majority of studies seem to suggest that dopamine strengthens the preference for higher valued, costly options.

## Relating Empirical Findings to Existing Accounts of Dopamine Functioning in Value-Based Choice

**Figure 1:**
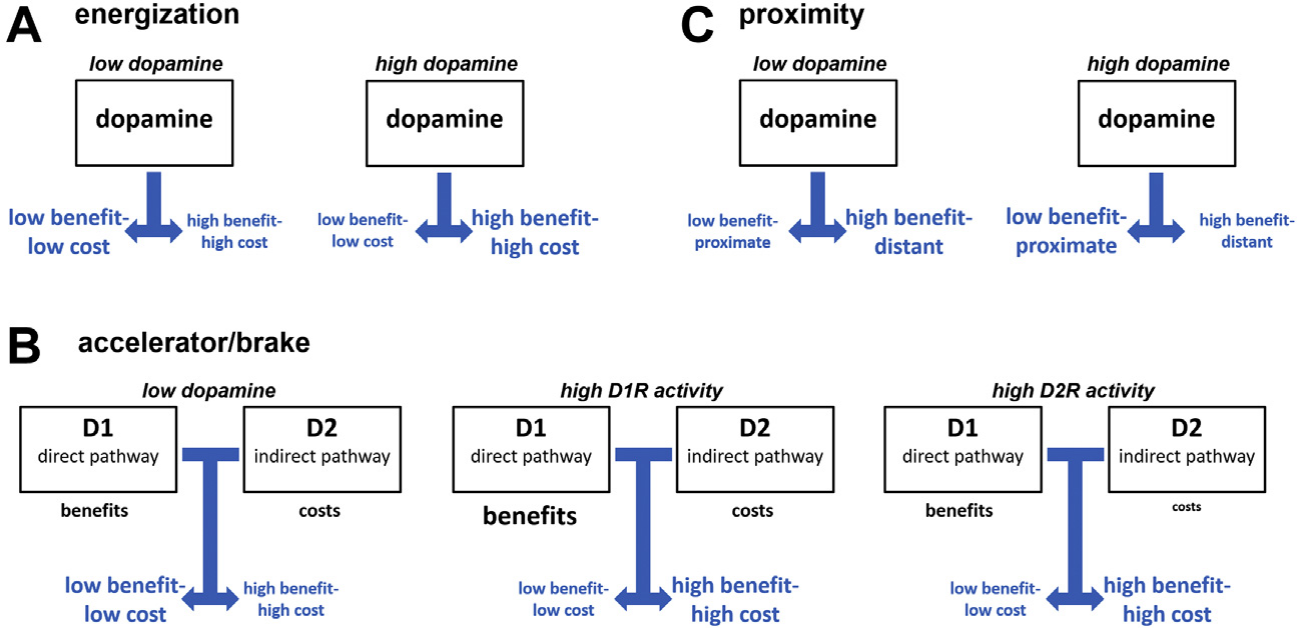
Prominent theories of dopamine function in value-based choice. **(A)** The energization account assumes that increased dopamine activation enhances the preference for high benefit–high cost options over low benefit–low cost options (illustrated by a larger font size for the high-cost compared with the low-cost option). **(B)** Building upon this notion, the accelerator/brake model posits that D_2_ receptor (D2R) activation computes the costs that are acceptable for a reward, which determines how strong a D_1_ receptor (D1R)–mediated facilitatory signal needs to be to overcome D2R-mediated inhibition. Thus, while both D1R and D2R activation strengthen the preference for high benefit–high cost over low benefit–low cost options, they do so via dissociable effects on processing of benefits vs. action costs. D1R activation strengthens the processing of rewards (larger font size), whereas D2R activation reduces the sensitivity to costs (smaller font size). **(C)** According to the proximity account, the impact of dopamine on behavior depends on the proximity advantage of the more proximate (in many cases, also the low cost) option over the more distant (often high cost) option. If one option has a proximity advantage over the other, dopamine strengthens the preference for the proximate over the distant reward, as illustrated here. In contrast, if no option possesses a proximity advantage, dopamine favors the high-benefit option in a similar way as assumed by the energization account.

In this section, we relate the empirical literature reviewed above to different theoretical accounts of the role of dopamine in cost-benefit decision making and consider how well these accounts explain the empirical data. An eminent account for the role of dopamine signaling in motivation is that dopamine energizes behavior toward goals (energization account; Figure 1A) (52,53). This account was based on findings that dopamine increases the willingness to work for rewards in rats (54, 55, 56, 57) and that (striatal) dopamine depletion abolishes speeding of reward-directed responses (58). It is also compatible with reports of increased dopamine release in the ventral striatum and ventromedial prefrontal cortex when rats respond more quickly because of higher reward rates (59). With regard to cost-benefit decision making, this account predicts that enhancing dopaminergic activity increases the preference for highly valued options despite the associated costs, while lower dopaminergic activity should decrease it. We note that different variants of this account have been formulated, some (60, 61, 62) also reconciling the motivational functions of dopamine with its involvement in reward learning (62,63) or ascribing dopamine a valence-independent role for approach behavior (64). Still, all the variants of this account link dopamine to choices of high reward–high cost options.

Regarding the reviewed literature, the energization account is consistent with the findings for effort-based choice, where increased dopaminergic activity enhances the willingness to tolerate effort costs. However, the energization account appears to be difficult to reconcile with the findings for D_2_ antagonists for risk and delay costs, because in these domains D_2_ antagonism increased, rather than decreased, the preference for high benefit–high cost options.

In contrast to the energization account, the accelerator/brake model (Figure 1B) distinguishes between the roles of different dopamine receptor subtypes and assumes that D1R and D2R neurotransmission enhance goal-directed behavior via separate computational mechanisms (65). While D1Rs in the direct path encode the benefit associated with an option (accelerator), D2Rs implement a cost control (brake) that needs to be overcome to suppress the inhibitory indirect path. If tonic dopamine levels are high (note that D2Rs are more sensitive to changes in tonic dopamine than D1Rs), even relatively small or costly rewards will elicit approach behavior. In contrast, a low tonus reduces the desirability of costly options, and only large above-average rewards will trigger reward seeking. This is also consistent with the view that tonic D2R-mediated dopaminergic activity encodes the background reward rate (66). Thus, the accelerator/brake account predicts D1R- versus D2R-targeting dopaminergic manipulations to have dissociable effects on the processing of benefits and costs in value-based decisions, with D1R activation increasing sensitivity to benefits and D2R activation lowering impact of action costs [similar to the assumptions of the opponent actor learning model (67)].

Evidence for dissociable roles of D1R and D2R is somewhat sparse, given that relatively few studies distinguished between effects on reward versus cost processing. Moreover, many studies analyze choice behavior with economic utility models, for example, hyperbolic delay discounting or prospect theory of risky choice. These models integrate rewards and action costs to quantify the utility of options and usually do not contain separate terms for costs and benefits. Among the studies distinguishing between cost and benefit processing, however, there is indeed some evidence that D1R activation shows stronger effects on reward than on cost processing, whereas manipulation of D2R neurotransmission changes cost sensitivity. As noted above, methylphenidate promotes decisions to engage in mental effort by strengthening the weight assigned to the potential benefits (37). While methylphenidate enhances both D1R and D2R activation, its impact on reward processing seems to be mediated primarily by D1R rather than D2R stimulation (68), suggesting that enhanced D1R activity may underlie the effects of methylphenidate on benefit processing in decision making. Evidence for D1R involvement in preferring costly higher rewards over cost-free lower rewards is also provided by a recent study with a selective D_1_ agonist (22). Relatedly, in a loss chasing task, the D_2_ antagonist pramipexole reduced costly attempts to recover losses (36), while methylphenidate increased sensitivity to high rewards (26). In addition, animal findings support the hypothesized role of D2R signaling for cost processing (69,70). These findings are consistent with the idea that D2R activity implements a cost control that determines how strong D_1_-mediated facilitatory signaling in the direct pathway needs to be to overcome D_2_-mediated indirect pathway suppression (65).

Finally, the proximity account (Figure 1C) highlights dopamine’s sensitivity to psychological proximity (71). According to this view, dopamine strengthens the preference for psychologically close (e.g., immediately available) over distant rewards. Evidence for proximity effects originally stems from animal findings showing enhanced firing rates of dopaminergic neurons for spatially close rewards (72), but Westbrook and Frank (71) extend the concept from physical proximity to psychological proximity. Although action costs and proximity may appear closely related, they can conceptually be distinguished: subjective costs are learned associations between actions and effort, waiting time, or risk and also vary depending on an agent’s internal state (e.g., available resources to exert effort). Proximity, in contrast, depends on situational factors such as the relative salience, familiarity, or concreteness of an option (71). Whereas in economic decision-making paradigms, costs and proximity are often confounded because less costly options are likely to be more proximate (e.g., outcomes in the near rather than far future are also more concrete), costs and proximity could in principle experimentally be distinguished (e.g., by making the future outcome more concrete; see Supplemental Discussion). The proximity account predicts that high dopaminergic activity strengthens the preference for proximate rewards compared with distant rewards. In contrast, if no option has a proximity advantage, dopamine favors high benefit–high cost actions, as also assumed by the energization and accelerator/brake models.

As tentative computational implementation of the proximity account (71), a proximity advantage shifts the starting point of the evidence accumulation in a drift diffusion model toward the more proximate option. Still, action costs and benefits can affect the drift rate (which captures the actual process of evidence accumulation) despite an initial starting bias, and the strength of these effects of costs and benefits on the accumulation process is mediated by receptor-specific dopaminergic activity. According to the proximity account (71), cost-benefit decisions depend on the interplay between dopaminergic effects on evidence accumulation for high benefit–high cost options and on an initial proximity advantage. Moreover, prefrontal mechanisms may increase thresholds, giving distant reward options more time to compete with options that are favored by a proximity advantage (73,74).

The proximity account is supported by intervention findings in intertemporal and risky choice where D_2_ antagonism increased the preference for delayed and risky outcomes. In addition, in the domain of social decisions, dopaminergic effects seem to differ between close and distant others (16,48), which may point to a potential proximity effect in prosocial giving where selfish rewards have a smaller proximity advantage over rewards shared with close than with distant others. In the domain of effort-based choice, however, there was no evidence for a proximity effect, and the result pattern rather consistently links dopamine with stronger preferences for high effort/high reward options. Accordingly, one may ask whether proximity effects are domain specific. In other words, why are delayed and risky rewards as well as rewards shared with strangers perceived as psychologically distant, whereas effortful rewards are not? A potential ad hoc explanation for this phenomenon is that in most effort-based decision paradigms, decision makers experience the required effort demands in a familiarization session where they learn that they can successfully deal even with high effort demands, such that all effort options are actually perceived as proximate (and potential confounds of effort aversion, such as risk aversion, are eliminated). Effortful rewards may therefore be perceived as just as psychologically close as effort-free rewards. Another possibility is that different dose-response curves underlie the mediating role of proximity in effort-based compared with intertemporal and risky choice. However, we emphasize that these explanations are speculative and depend on several assumptions (e.g., whether a given compound in a given dose acts primarily pre- or postsynaptically), such that they need to be tested by future studies.

## Toward a Unifying Account of Dopamine in Value-Based Choice

**Figure 2:**
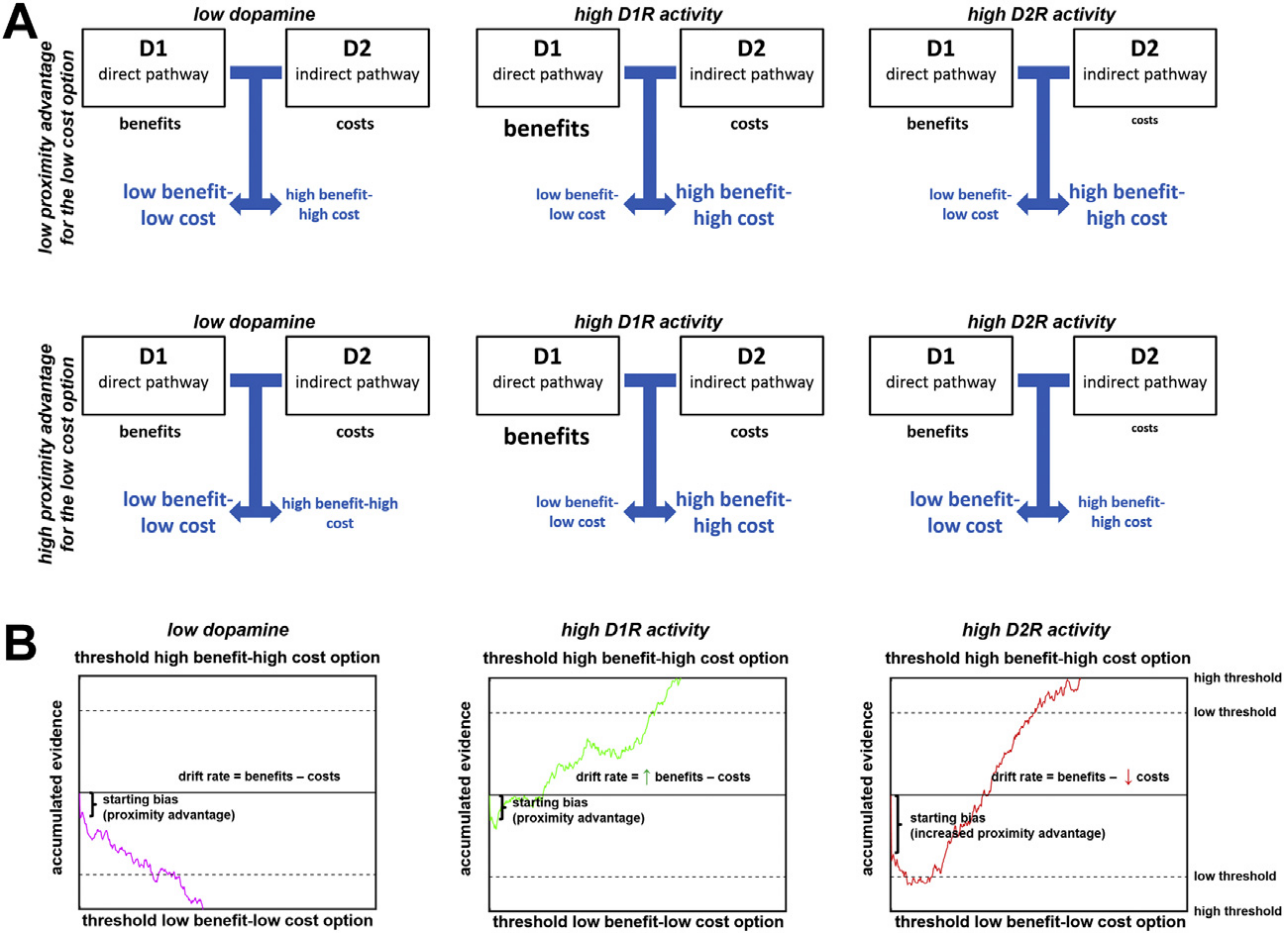
Toward a unifying account of dopamine in value-based choice. **(A)** Combining the accelerator/brake with the proximity model (Figure 1), we propose that dopamine affects cost-benefit decision making both by enhancing the proximity advantage of proximate low-cost (e.g., sooner rewards) over distant high-cost options (e.g., later rewards) and by implementing a cost control. If no option possesses a proximity advantage over the other (upper panel), our account makes the same predictions as the accelerator/brake account, i.e., increasing both D_1_ receptor (D1R) (via enhanced benefit processing) and D_2_ receptor (D2R) activity (by increasing the acceptable costs) enhances the preference for high-benefit rewards. If the low-cost option is also more proximate than the high-cost option (lower panel; note that costs and proximity can conceptually be distinguished), low dopamine levels (by reducing the influence of the proximity advantage) and increased D1R activity strengthen the preference for more distant–high benefit rewards. In contrast, high D2R activity enhances both the proximity advantage of the more proximate (low cost) option and the acceptable costs. **(B)** Within the framework of a drift diffusion–style model of the choice process, D2R activation might increase the proximity advantage of low-cost options by shifting the starting point of the evidence accumulation process toward the boundary of the low-cost option (71). During evidence accumulation, D1R activation strengthens the impact of benefits on the velocity of the accumulation process (drift rate), while higher D2R activation lowers the sensitivity to action costs. Thus, if decision thresholds are low, stronger proximity effects under high D2R activation increase the likelihood of choosing the (proximate) low-cost option, whereas in cases of high decision thresholds, D2R effects on evidence accumulation will result in more choices of the high benefit–high cost option.

Combining the accelerator/brake account with the proximity approach (Figure 2A) may provide a unified account of dopamine’s role in decision making. First, if a choice option possesses a proximity advantage (i.e., temporally close or low-risk rewards) over alternative options, higher tonic dopamine levels favor proximate over distant rewards. From the perspective of process models, the advantage corresponds to an initial bias toward the proximate option before the evidence accumulation process begins (71). Assuming that this bias is D_2_-mediated explains why D_2_ antagonists increase the willingness to tolerate risk and delay costs (15, 16, 17, 18,34, 35, 36). In the absence of consistent evidence that D1R-stimulating drugs increase the preference for proximate (i.e., risk-free or immediate) rewards, the reviewed literature (8, 9, 10,20,22,27) does not suggest a role of D1Rs in moderating proximity effects due to lack of consistent evidence that D1R-stimulating drugs increase the preference for proximate (i.e., risk-free or immediate) rewards. We therefore posit that the proximity bias is (preferentially) moderated by D2R rather than D1R activation, but note that additional empirical work is needed to directly test this assumption.

Second, D2R activation releasing the inhibitory impact of the indirect path may implement a cost control that determines whether a benefit is considered worth its costs (65). If tonic dopamine levels (and thus D2R activation) are low, the indirect path exerts a strong inhibitory influence on the cortex, such that only large rewards will lead to sufficient D1R-mediated direct path facilitation to overcome the D2R-mediated inhibition. In contrast, high tonic dopamine releases the inhibitory impact of the indirect path, such that relatively small rewards also become a worthy pursuit (22,26, 27, 28, 29, 30,37, 38, 39, 40). Computationally, this may be implemented through D1R and D2R activation affecting evidence accumulation rather than the starting bias. Once the evidence accumulation process has started (which trades the benefits against the costs of action alternatives), D1R and D2R mediate evidence accumulation for benefits and costs, respectively, associated with the action alternatives. In this way, dopamine can promote the choice of high benefit–high cost options despite an initial proximity advantage of low-cost rewards, particularly if decision thresholds are high and agents make cautious choices. The assumption that proximity effects are moderated by D2Rs rather than D1Rs makes the straightforward prediction that changes in decision thresholds may reverse the influence of manipulations of D2R activation on observed choices (Figure 2B). This account thus integrates several aspects of the empirical data that cannot be explained by existing accounts in isolation. The impact of D_2_ antagonists on intertemporal and risky choice cannot be explained by the energization or accelerator/brake accounts, unless one implausibly assumes that all administered D_2_ antagonists only have presynaptic rather than postsynaptic effects. Moreover, the dissociable effects of D1R and D2R activity on benefit versus cost processing were unspecified in the current formulation of the proximity model, which includes no explicit predictions for separate contributions of receptor types to the choice process (the focus seems to be on the role of tonic dopamine for moderating proximity effect). The proposed model therefore specifies the proximity account by more strongly emphasizing the dissociable roles of D1Rs and D2Rs for the choice process. As a caveat, we note that the focus of our account is primarily on striatal dopamine, but dopamine receptors are also expressed in other brain regions such as the prefrontal cortex, where D1Rs outnumber D2Rs (75). Because prefrontal D1R activation increases goal representations (76), striatal and prefrontal D1Rs may have similar effects on observable behavior. Nevertheless, given that the reviewed pharmacological manipulations are systemic, it remains open whether the drug-induced behavioral changes are mediated via effects on striatal or prefrontal receptor activity.

The proposed account predicts that the height of the decision threshold crucially influences the impact of dopamine on choice behavior: if decision thresholds are low (and agents make impetuous decisions), dopaminergic effects on the proximity advantage dominate, increasing choices of proximate, low-cost rewards. In contrast, under high decision thresholds (cautious decisions), dopaminergic effects on evidence accumulation may win out over initial proximity advantages, resulting in a higher likelihood of high benefit–high cost choices. Such a model of the role of dopamine for value-based choice allows integrating a large body of empirical evidence, which appeared partially inconsistent with previous existing accounts. The hypothesized crucial role of decision thresholds may also deepen our understanding of clinical decision-making deficits, given evidence from computational psychiatry for altered thresholds in some disorders (77,78). Increased thresholds in depression (77) lower the impact of proximity advantages on choices, which may explain why lower dopamine levels in depression lead to stronger delay discounting (79). In contrast, the positive correlation between delay discounting and dopamine levels in ADHD (80) can be explained by a lower decision threshold (81), which leads to a more influential role of dopamine effects on proximity advantages. Our account may thus explain why the link between dopamine and impulsivity varies between clinical and nonclinical populations (80). While details of the model (particularly its computational implementation) may change with growing empirical evidence, its principles represent a fruitful hypothesis that may put the heterogeneous field of dopaminergic studies in human decision making on more solid theoretical and computational grounds. We note that the computational role of dopamine in decision making has already been formalized previously (67,82,83). Therefore, a valuable enterprise for future empirical studies would be to directly test the predictions of these different accounts.

We close by noting that the lack of a unifying account of dopamine in decision making is at least partially caused by limitations in study designs and inherent properties of the biological substrate that hamper theory development. First, it is often unclear whether an effect of a pharmacological challenge reflects increased or decreased dopaminergic activation. For example, in studies using agonists, a drug-induced change in behavior may either reflect increased functioning relative to baseline or—assuming an inverted u–shaped dopamine-response curve (84)—reduced functioning due to increased dopaminergic activation beyond optimal dopamine levels. To make matters worse, evidence suggests that the shape of the dopamine-response curve is domain specific (22,84,85). To clarify the direction of effects in pharmacological intervention studies, we therefore recommend using multiple doses and measuring baseline dopamine levels, either directly (via positron emission tomography) or indirectly [e.g., with working memory performance (86) or trait impulsivity (87); see (21,22,85)]. An additional issue exists for D_2_ antagonists (such as amisulpride, sulpiride, or haloperidol), which, depending on the administered dose, can either increase or decrease dopaminergic activity through presynaptic or postsynaptic actions, respectively (88). For safety reasons, many studies administer a dose at the lower border for postsynaptic effects, which again hinders a straightforward interpretation of the direction of the effects.

Besides these methodological considerations, a further recommendation relates to statistical analysis where many previous studies did not explicitly distinguish between reward- versus cost-mediated effects. When assessing aggregated mean choice behavior or parameters from economic utility models integrating costs and rewards to a subjective value term, it is not possible to test for potential receptor-specific contributions to reward and cost processing. A way to address this issue is to analyze the effects of pharmacological interventions on decision making on attribute-wise comparisons of reward magnitudes and costs (e.g., the delay of the smaller-sooner and the larger-later option in intertemporal choice). Such attribute-wise comparisons were recently found to explain choice behavior better than comparisons between integrated subjective values (89,90). Moreover, they allow testing of the hypothesized attribute-specific contributions of different receptor subtypes. Finally, to test the hypothesis of an early dopamine-mediated proximity effect (71), we recommend employing process models such as the drift diffusion model, that provide insights into subcomponents of the decision process by integrating choice and decision time data (17,37).

Together, these recommendations for improving study designs and statistical analyses will conceptually advance research on dopamine and decision making by assessing central assumptions of the model we proposed (although evidently not all future studies need to fulfill all of these criteria to produce valuable insights). Such a unifying account is not only of theoretical value but will also deepen our understanding of the biological causes of decision-making deficits in psychiatric disorders and improve predictions for the behavioral effects of pharmacological treatments. Integrating the notions that dopamine implements a cost control and is sensitive to proximity differences between action options, the proposed model has the potential to advance and unify our mechanistic understanding of cost-benefit decision making in healthy and clinical populations.
