# Supplementary material for: Toward a Unifying Account of Dopamine’s Role in Cost-Benefit Decision Making
Source: Biol Psychiatry Glob Open Sci. 2022 Mar 8;3(2):179–86. doi: 10.1016/j.bpsgos.2022.02.010 (PMC10140448; doi:10.1016/j.bpsgos.2022.02.010)
Supplement: Supplementary Material [file mmc1.pdf]

Supplementary Information for

Towards a Unifying Account of Dopamine's Role in Cost-Benefit Decision Making

Mathematical formulation of the proposed framework

We propose that dopamine affects different components of the choice process, which can be modelled as a drift diffusion-style process. Drift diffusion models assume that, after a non-decision time τ, evidence is accumulated with a velocity (drift rate) ν from a starting bias ζ until a decision threshold α is reached. Following the assumptions of the OpAL or accelerator/brake models (1), we assume that the drift rate towards the boundaries depends on the linear combination of the differences in reward magnitude ($Magnitude_{diff}$) and action costs ($Cost_{diff}$) between two choice options:

$$\nu = \beta_{magnitude} \times Magnitude_{diff} - \beta_{cost} \times Cost_{diff}$$

According to the proximity account (2), the starting bias parameter is sensitive to the proximity advantage of one option over the other ($Proximity_{diff}$), which can be formalized as follows:

$$\zeta = \beta_0 - \beta_{proximity} \times Proximity_{diff}$$

We posit that dopaminergic D1R and D2R activity affects different subcomponents of this choice process, making clear predictions regarding which aspect of the choice process should be affected by a drug as a function of its effects on D1R and D2R family activation. D1R activation determines the weight assigned to rewards in the evidence accumulation process ($\beta_{magnitude}$). In contrast, D2R activation affects the weight assigned to both action costs during evidence accumulation (with higher D2R activation reducing β_{cost}) and to proximity effects on the starting bias (with higher D2R activation increasing $\beta_{proximity}$). Analyzing existing or novel

datasets with drift diffusion models allows empirically testing these assumptions and modifying the proposed framework according to the empirical findings.

We note that another model of the authors of the proximity account assumes that D1-mediated benefit processing is weighed against D2-mediated cost processing ((3); see also equation 1 in (2)), but in the formulation of the proximity account the idea that D2R activation computes the acceptable costs appears to have been crowded out by the focus on proximity. In their normative account of how manipulating dopamine levels affects value-based choice, the authors of the proximity account do not explicitly assume distinct roles for D1R and D2R neurotransmission. Given the evidence for dissociable influences of D1R and D2R activation on decision making, we propose that combining the assumptions of a D2-mediated cost control with proximity-dependent dopaminergic effects may provide the best explanation for the role of dopamine in cost-benefit decision making, as we describe in the main text.

Disentangling proximity and action costs

In most situations, action costs and proximity differences are strongly correlated. In intertemporal decision making, for example, immediate rewards are also perceived as more proximate than delayed rewards. However, it is possible to experimentally disentangle proximity and action costs by manipulating the concreteness of the delayed reward, e.g. via episodic event cues. In some previous experiments, a reward in the future was presented either with or without an episodic cue for an event occurring at the time of reward delivery (e.g., "vacation in Paris in 180 days"; Figure S1A/B) (4-6). These episodic event cues increased the proportion of larger-later choices, which was explained by the assumption that the event cues make the future rewards more concrete. In other words, these event cues reduced the proximity advantage of the immediate over the delayed reward without affecting the action costs (as the waiting time stayed the same). If this assumption is correct, then from the perspective of process models the more patient choices in conditions with compared to without episodic event cues

should result from a shift in the starting bias towards the delayed reward. In contrast, the weighted influences of rewards and delay costs on the evidence accumulation process per se should be unaffected. Thus, episodic events cues, which render future or also risky outcomes more concrete (5), provide a tool for disentangling proximity and action costs. Manipulations like these may even allow assigning a proximity advantage to the costlier option if the difference in action costs between the options is small (Figure S1C).

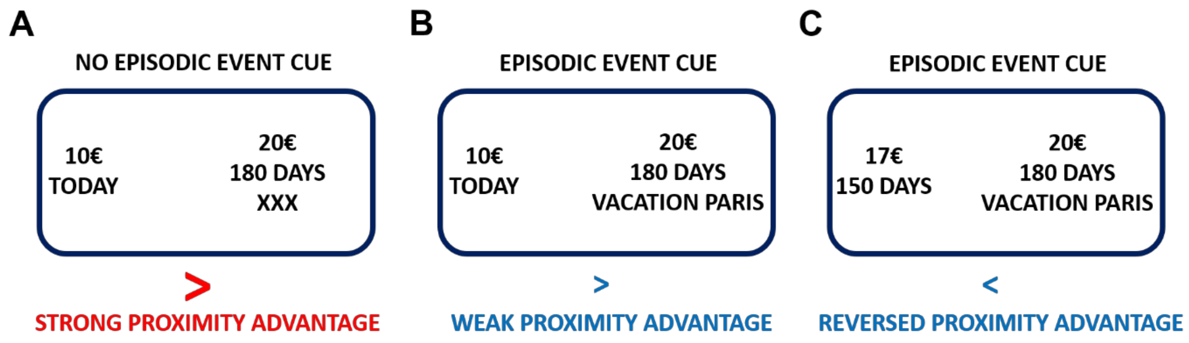

Figure S1. Experimentally manipulating proximity bias. (A) In most experimental paradigms less costly (e.g., immediate) rewards have a proximity advantage over costlier (e.g., delayed) rewards. However (B), this proximity advantage can be reduced by making the delayed option more concrete, for example via episodic event cues. (C) If differences in action costs are sufficiently small (e.g., slightly less reward in 150 days versus in 180 days), such event cues may even allow reversing the proximity bias and assigning a proximity advantage to the costlier over the less costly reward.

Supplementary References

1. Beeler JA, Mourra D (2018): To do or not to do: dopamine, affordability and the economics of opportunity. *Frontiers in integrative neuroscience*. 12:6.
2. Westbrook A, Frank M (2018): Dopamine and Proximity in Motivation and Cognitive Control. *Curr Opin Behav Sci*. 22:28-34.
3. Collins AG, Frank MJ (2014): Opponent actor learning (OpAL): modeling interactive effects of striatal dopamine on reinforcement learning and choice incentive. *Psychological review*. 121:337-366.
4. Peters J, Buchel C (2010): Episodic future thinking reduces reward delay discounting through an enhancement of prefrontal-mediotemporal interactions. *Neuron*. 66:138-148.
5. Mok JN, Kwan D, Green L, Myerson J, Craver CF, Rosenbaum RS (2020): Is it time? Episodic imagining and the discounting of delayed and probabilistic rewards in young and older adults. *Cognition*. 199:104222.
6. Bulley A, Gullo MJ (2017): The influence of episodic foresight on delay discounting and demand for alcohol. *Addictive behaviors*. 66:1-6.

Supplementary Tables

Table S1. Effects of dopaminergic manipulations on decisions involving trade-offs between rewards and delay costs.

First author	Year	N	Design	Drug	Dose	Receptor	Action	Outcome
De Wit	2002	36	Within	d-amphetamine	10 mg<br>20 mg	D1/D2	agonist	less delay discounting under 20 mg dose
Kayser	2012	23	Within	tolcapone	200 mg	D1/D2	agonist	less delay discounting
Acheson	2008	32	Within	d-amphetamine	20 mg	D1/D2	agonist	no effect
				bupropion	150 mg<br>300 mg	D1/D2	agonist	no effect
Soutschek	2020	120	Between	PF-06412562	6 mg<br>15 mg<br>30 mg	D1	agonist	no effect
Pine	2010	13	Within	l-dopa	150 mg	D1/D2	agonist	stronger delay discounting
				haloperidol	1.5 mg	D2	antagonist	no effect
Kelm	2013	15	Within	APTD		D1/D2	antagonist	no effect
Hamidovic	2008	10	Within	pramipexole	0.25 mg<br>0.5 mg	D2	agonist	no effect
Arrondo	2015	14	Within	metoclopramide	10 mg	D2	antagonist	less delay discounting
Soutschek	2017	55	Within	amisulpride	400 mg	D2	antagonist	less delay discounting
Weber	2016	81*	Between	amisulpride	400 mg	D2	antagonist	less delay discounting
Wagner	2020	54	Between	haloperidol	1.5 mg	D2	antagonist	less delay discounting
Petzold	2019	87	Within	l-dopa	150 mg	D1/D2	agonist	no effects

Action can be direct (through binding at receptors) or indirect, e.g. by blocking reuptake.
*The study included an additional group of 40 participants who received the opioid receptor antagonist naltrexone.
APTD: acute phenylalanine/tyrosine depletion method.

Table S2. Effects of dopaminergic manipulations on risky decision making.

First author	Year	N	Design	Drug	dose	Receptor	Action	Outcome
De Wit	2002	36	Within	d-amphetamine	10 mg<br>20 mg	D1/D2	agonist	no effect
Rigoli	2016	32	Within	l-dopa	150 mg	D1/D2	agonist	more risky decisions
Acheson	2008	32	Within	d-amphetamine	20 mg	D1/D2	agonist	no effect
				bupropion	150 mg<br>300 mg	D1/D2	agonist	no effect
Soutschek	2020	120	Between	PF-06412562	6 mg<br>15 mg<br>30 mg	D1	agonist	fewer risky choices with increasing dose
Rutledge	2015	30	Within	l-dopa	150 mg	D1/D2	agonist	more risky decisions for gains
Evers	2017	24	Within	methylphenidate	40 mg	D1/D2	agonist	no effects
Symmonds	2013	20	Within	l-dopa	100 mg	D1/D2	agonist	no effect
Hamidovic	2008	10	Within	pramipexole	0.25 mg<br>0.5 mg	D2	agonist	no effect
Arrondo	2015	14	Within	metoclopramide	10 mg	D2	antagonist	no effect
Burke	2018	93	Between	amisulpride	400 mg	D2	antagonist	less risk aversion and probability distortion
Ojala	2018	21	Within	sulpiride	400 mg	D2	antagonist	less probability distortion
Riba	2008	15	Within	pramipexole	0.5 mg	D2	agonist	more risky choices
Petzold	2019	87	Within	l-dopa	150 mg	D1/D2	agonist	fewer risky choices in impulsive individuals
White	2007	37	Within	d-amphetamine	20 mg	D1/D2	agonist	more risky choices in individuals with high reward sensitivity
Campbell-Meiklejohn	2012	40	Between	methylphenidate	20 mg	D1/D2	agonist	increased sensitivity to high rewards
Campbell-Meiklejohn	2011	40	Between	pramipexole	0.176 mg	D2	antagonist	reduced loss chasing
Norbury	2013	20	Within	cabergoline	1.5 mg	D2	agonist	higher sensitivity to probability of winning
Zack	2007	18	Within	haloperidol	3 mg	D2	antagonist	no effect
Gross	2021	154	Between	methylphenidate	30 mg	D1/D2	agonist	more risky choices

Action can be direct (through binding at receptors) or indirect, e.g. by blocking reuptake.

Table S3. Effects of dopaminergic manipulations on decisions involving trade-offs between rewards and effort costs.

First author	Year	N	Design	Drug	dose	Receptor	Action	Outcome
Soutschek	2020	120	Between	PF-06412562	6 mg<br>15 mg<br>30 mg	D1	agonist	more high effort choices with increasing dose
Wardle	2011	17	Within	d-amphetamine	10 mg<br>20 mg	D1/D2	agonist	more high effort choices under 20 mg dose
Westbrook	2020	50	Within	methylphenidate	20 mg	D1/D2	agonist	more high effort choices
				sulpiride	400 mg	D2	antagonist	more high effort choices
Zenon	2016	19	within	l-dopa	125 mg	D1/D2	agonist	more high effort choices
Michely	2020			l-dopa	150 mg	D1/D2	agonist	higher motivation to exert effort
				haloperidol	1.5 mg	D2	antagonist	less strategic effort discounting
Cawley	2013	32	Within	APTD		D1/D2	antagonist	lower motivation to exert effort
Dean	2016	17	Within	bupropion	150 mg	D1/D2	agonist	no effect
Ohmann	2020	203	Between	sulpiride	200 mg	D2	antagonist	less effort exerted
Korb	2020	131	Between	amisulpride	400 mg	D2	antagonist	less effort exerted

Action can be direct (through binding at receptors) or indirect, e.g. by blocking reuptake.
APTD: acute phenylalanine/tyrosine depletion method

Table S4. Effects of dopaminergic manipulations on prosocial decision making.

First author	Year	N	Design	Drug	dose	Receptor	Action	Outcome
Soutschek	2017	55	Within	amisulpride	400 mg	D2	antagonist	fewer prosocial choices in females<br>more prosocial choices in males
Saez	2015	35	Within	tolcapone	200 mg	D1/D2	agonist	stronger inequity aversion
Pedroni	2014	197	Between	l-dopa	300 mg	D1/D2	agonist	fewer prosocial choices in males
Oroz-Artigas	2019	33	Within	pramipexole	0.35 mg	D2	agonist	fewer prosocial choices in females
Crockett	2015	86	Between	l-dopa	150 mg	D1/D2	agonist	fewer prosocial choices
Gross	2021	154	Between	methylphenidate	30 mg	D1/D2	agonist	no effect

Action can be direct (through binding at receptors) or indirect, e.g. by blocking reuptake.
